# Analysis of the Experiences of Adults with Complex Regional Pain Syndrome

**DOI:** 10.3390/healthcare9070878

**Published:** 2021-07-13

**Authors:** Soo Jin Kwon, Yoonjung Kim

**Affiliations:** 1 Department of Nursing, Ansan University, 155 Ansandaehak-ro, Sangrok-gu, Ansan-si 15328, Korea; soojinyk@gmail.com; 2 Red Cross College of Nursing, Chung-Ang University, 84 Heukseok-ro, Dongjak-gu, Seoul 06974, Korea

**Keywords:** complex regional pain syndrome, nursing, pain measurement, qualitative research

## Abstract

Complex regional pain syndrome is a rare, intractable disease causing chronic pain. For improved subjective and personal experience, an individualized treatment approach based on a thorough understanding of the patient’s perceptions is required for pain management. In this study, we examined the experiences and challenges of 11 Korean patients diagnosed with complex regional pain syndrome. The patients described their experiences during in-depth, face-to-face interviews, and data were subjected to a thematic analysis. We identified the following three main themes: “my own non-stereotyped pain,” “complex emotions caused by pain,” and “a careful life endured alone.” Enduring pain alone was difficult, and the lack of support from family members, caregivers, or society amplified the patients’ hardships. As these patients often felt alone when coping with internal difficulties, including pain, they frequently coped through self-management of the condition. The importance of offering realistic support to complex regional pain syndrome patients is underscored via a multifaceted approach and may aid in the development of educational programs for medical personnel, families, and caregivers of these patients.

## 1. Introduction

Complex regional pain syndrome (CRPS) is a rare, intractable disease characterized by chronic pain. The condition presents with inflammatory changes and signs at sites of pain, changes in skin color and temperature, sweating, severe hyperalgesia and allodynia, edema, altered patterns of hair and nail growth, reduced strength, dystonia, and tremors [1]. The International Association for the Study of Pain agreed on the term CRPS to describe this unique disorder characterized by spontaneous or stimulus-induced pain [2].

The diagnosis and treatment of CRPS are difficult. As CRPS is associated with several causes and diverse clinical presentations, the disease etiology is not fully understood [1]. Thus, CRPS is diagnosed based on clinical signs and symptoms, making it challenging for both medical personnel and patients [3,4]. Late diagnosis of CRPS can lead to long-term distress and, in some cases, unnecessary, invasive debridement procedures [5]. Thus, medical providers must recognize the various states of CRPS and use a multidimensional treatment approach without preconceptions; otherwise, failure to accurately recognize CRPS jeopardizes the validity of patients’ symptomatology [6]. There is limited knowledge about the mechanisms involved in CRPS, and current treatments rely mainly on the efficacy observed in other common neuropathic pain conditions [7]. Treatment options for CRPS include physical and occupational therapy; administration of bisphosphonates, calcitonin, subanesthetic intravenous ketamine, free radical scavengers, and oral corticosteroids; and spinal cord stimulation [4].

The incidence of CRPS is 26.2 per 100,000 person-years in the Netherlands [8] and 29.0 per 100,000 person-years in Korea [9]. As CRPS is a rare disease, it is difficult to compare study findings or conduct reliable, large-scale, randomized controlled trials [10]. YMoreover, in Korea, 80% of patients diagnosed with CRPS are unemployed, and CRPS itself has negative effects on financial circumstances and quality of life [11]. Therefore, CRPS patients may be affected psychologically and socially, and for a successful recovery of patients, a multifaceted approach and research are required along with fast and appropriate treatment.

To date, research on CRPS has explored the disease’s diagnosis, causes [12,13,14], and treatment [15,16]; the utility of a psychosocial treatment approach [17,18]; and patients’ experiences after participating in a rehabilitation program [19]. Qualitative research regarding distorted body perception in patients with CRPS has also been conducted [20]. As limited research on CRPS has been conducted in Korea, there is a lack of clinical understanding regarding the subjective experiences of patients with CRPS in the country [9,11]. Ascertaining the subjective meaning of pain in relation to sociocultural factors may help in better understanding the disease. Therefore, the aim of the study was to examine the experiences and challenges of Korean adult patients with CRPS.

## 2. Materials and Methods

### 2.1. Design

This study was a thematic analysis of data collected through individual, in-depth interviews of adult patients diagnosed with CRPS.

### 2.2. Participants

The study included six men and five women aged 20–65 years who had been diagnosed with CRPS at a university hospital in accordance with the International Association for Study of Pain criteria. Eight patients were married. The most common cause of CRPS was reported as “after a road traffic accident” (six participants; Table 1). To check the level of the participants’ usual pain, pain was expressed using a numeric rating scale. A score of 0 meant no pain, and a score of 10 meant unbearable pain; the higher the number, the more severe the pain. In participants with multiple pain areas or whose pain level changed frequently, it was difficult to express the exact level of pain. For such patients, the approximate score was expressed as the average pain score.

### 2.3. Ethical Considerations

Patients were recruited through posters displayed in the hospital outpatient department, and all patients provided written informed consent for study participation. This study was approved by the institutional review board of the hospital (H-1106-122-368) where the research was conducted. Before the interviews, the participants were provided written and oral explanations of the research objectives and methods and asked to provide written informed consent for participation. Participants were informed that the interview recordings would be used only for research purposes and that observations or recordings from the interviews may be quoted in this research and that their anonymity would be guaranteed by not quoting potentially identifiable personal information. Participants were free to refuse or terminate participation at any time, including during the interview or if the pain was making it difficult for them to continue the interview. We made every effort to protect the participants from any mental or physical discomfort that might arise due to the interview.

### 2.4. Data Collection

Data were collected between October 2011 and May 2013. We conducted an in-depth, non-structured interview to encourage participants to speak honestly and openly about their own disease experiences. Interviews were conducted by one of the authors at a hospital in an outpatient consultation room.

The interview was completed in 60–90 min (in consideration of the participants’ pain and condition). Participants were informed that they could stop the interview at any time if they felt discomfort due to pain, but most participants did not stop the interview. Participants who experienced severe pain during the interview showed a pattern of frequent resting between words.

The interview questions were semi-structured, and the core question was, “What have your experiences as a patient with CRPS been like?” Specific questions included the following: “What were your thoughts and experiences after you were diagnosed?”, “What were the effects of the disease and treatment?”, “What have you experienced between the diagnosis and now?”, “How have you dealt with the disease?”, and “What has affected your experience of the disease?” The researchers encouraged participants to describe their experiences completely. During the interview, researchers noted any non-verbal responses or other observed details that they considered important. The recorded interview content was transcribed for analysis.

### 2.5. Data Analysis

We performed a thematic analysis based on the method of Braun and Clarke [21], focusing on the significance of common themes. This thematic analysis method is divided into six phases. Phase 1, familiarization with data, involved reading through written data multiple times to identify significant content regarding the difficulties and experiences of participants in relation to CRPS symptoms. In Phase 2, initial code generation, 230 significant statements were extracted and coded. In Phase 3, searching for themes, codes were organized in terms of provisional themes, all data relating to the provisional themes were collected, and 45 provisional themes were derived. Phase 4, reviewing the themes, involved identifying whether the extracted themes were consistent with the overall data. Here, we ultimately derived three themes and six subthemes. In Phase 5, naming the themes, the themes were named, and their meanings were defined. In Phase 6, report writing, we generated a final review of the contents and results of the study.

We examined four criteria to validate the trustworthiness of this qualitative study, as suggested by Lincoln and Guba [22], namely credibility, transferability, dependability, and repeatability. Researchers conducted the study in accordance with qualitative research methods, aiming to exclude bias and maintain neutrality throughout all stages of the study. The derived themes and subthemes were checked by two researchers, and data analysis and derivation of results were conducted under the guidance of a nursing professor with experience in qualitative research.

## 3. Results

### 3.1. Theme 1: My Own Non-Stereotypical Suffering

The pain experienced by each patient differed in the pattern, intensity, and cause, but most patients reported experiencing severe pain that was difficult to tolerate. Because this pain was not stereotypical, patients had often endured a difficult period until CRPS was recognized and diagnosed. As such, patients perceived their condition as a form of personal suffering. Although the pain became familiar and the intensity of pain decreased over time, it did not disappear in any of the patients.

#### 3.1.1. Fear of the Unique Presentation and Extremely Severe Pain 

Although there was diversity in the patterns, intensity, frequency, duration, and aggravating/alleviating factors for pain, most patients described the pain as extremely severe and unbearable. Patients consistently described CRPS symptoms as unusual pain unlike any that they had previously experienced. They said that the pain made them feel afraid.

“It feels like touching a glass shard that’s buried beneath my skin. And what would I do then? That fear keeps me awake at night.”

(Participant 3)

“Lately, about once a day, I feel a sudden pain that climbs up my leg. It’s so bad that I can’t even move. I don’t know how I’ll survive if that sensation keeps coming …”

(Participant 4)

“When the pain comes, I can’t bear it; I bent over on the spot and started screaming. Because it hurts … I don’t want people to see me like that… (*omitted*) That’s why I’m thinking about taking leave from school again.”

(Participant 6)

#### 3.1.2. Gradually Getting Used to My Own Pain

At first, patients suffered from their pain and spent time searching for different treatment methods. Yet, in the end, treatments were ineffective, and the patients found that there was no specific treatment method available. As patients became accustomed to the uniqueness of the pain, they started recognizing its patterns and the aggravating and alleviating factors. Over time, they were better able to anticipate the pain to some extent, resulting in steady improvements in pain intensity and duration. Patients reported that they became more familiar with their pain over time.

“It’s better than it used to be. If it was strong-to-moderate before, now it’s moderate-to-light.”

(Participant 10)

“I received all kinds of treatments. I tried nerve blocks, medication …, but none of it was of any use … (omitted) … It’s a lot of pain, but in the end, I have to get through it by myself. I do feel like I’m getting used to it more and more.”

(Participant 3)

### 3.2. Theme 2: Complex Emotions Caused by Physical Pain

The fact that family members, doctors, and other individuals did not understand their pain caused physical and psychological hardship in patients. Patients showed a strong desire for acknowledgment, understanding, and compensation for their pain and distress. Yet, the preconception of CRPS being a rare, intractable disease caused patients to be conscious of others’ attention and therefore tried to hide their diagnosis.

#### 3.2.1. Secondary Emotional and Mental Distress 

These patients reported experiencing suffering alone due to severe physical pain that was not understood, acknowledged, or compensated by others. The lack of clear methods for the diagnosis and treatment of CRPS caused complex emotions and psychological problems, such as anger, shock, surprise, stress, fear, disappointment, despair, and helplessness. Therefore, patients showed secondary mental health problems resulting from their physical pain.

“I cried for days after receiving the diagnosis. I couldn’t believe that I had a rare disease and had become a cripple …”

(Participant 2)

“At first, I didn’t know what this disease was. But then I saw a TV program about the disease … It was awful… I was scared that there wasn’t really any way to treat it.”

(Participant 4)

“To be honest, I was shocked when I learned what disease it was. I couldn’t sleep, and I took antidepressants.”

(Participant 6)

“It’s not covered by insurance, and no one knows my pain. When I go to the hospital, I’m in pain, but sometimes they tell me I’m not … My family doesn’t know how much I’m hurting either.”

(Participant 7)

#### 3.2.2. The Conflicting Desire to Hide the Fact That I Am a Patient with CRPS 

Although patients showed a strong desire to have their pain acknowledged and understood, they also showed a conflicting desire to hide their disease due to misguided preconceptions about CRPS. In other words, although the patients reported wanting others to recognize their pain and suffering, they ultimately acknowledged that no one would be able to solve their problems and that others were likely to have negative preconceptions about CRPS.

“At first, I felt angry and unfairly treated because no one knew my pain … (omitted) … And the people who do know think this disease is so serious that you can’t move at all. Other people say that it’s not a common disease … They look at you like you’re abnormal, so I prefer not to say anything.”

(Participant 6)

“Because I feel like people think I’m strange. I try not to tell anyone as much as possible and to keep a low profile. I tend to worry a lot.”

(Participant 10)

### 3.3. Theme 3: Living Carefully, Enduring Alone

Patients with CRPS described different ways of coping with their pain, whether they continued to fight the disease, gave up and accepted the disease, or learned to control the disease themselves. As patients recognized that no one understood their pain and that there are no effective treatment methods, they persisted alone and endured a careful life trying to avoid pain.

#### 3.3.1. Gradually Learning My Own Way of Coping

Initially, patients showed proactive coping behavior by trying different methods to treat or alleviate their pain. Yet, as patients experienced various complications of treatment and related secondary problems, they often abandoned the idea of treatment and eventually accepted that there was no clear, precise treatment method for CRPS. Patients gradually learned their own means of coping by making efforts to control the pain themselves or by depending on somebody else.

“At first, I received analgesic injections, I had a cast, I tried physiotherapy … (omitted) … I tried taking pain medication, but I still feel a dull pain. It doesn’t stop the pain; it just dulls it … I feel a little zoned out … (omitted) … There is no real solution. Just keeping yourself warm.”

(Participant 4)

“This disease feels a little better when I’m emotionally at peace. So, I just try to live my life without worrying.”

(Participant 2)

“My mother has helped me a lot. That’s because she’s a teacher, so she understands students’ state of mind. I depend on my mother.”

(Participant 5)

#### 3.3.2. Always Living Carefully 

Patients reported experiencing a time of distress before learning that CRPS was poorly understood and largely untreatable. They endured pain while learning their own coping methods and led a constantly careful life. Although the patients depended significantly on the help or support of their family, they said that they ultimately understood that they themselves had to endure the pain and make careful judgments to avoid causing further pain. Therefore, they changed their lifestyles and led careful lives.

“I became incredibly sensitive. I am surprised whenever something touches my skin … (omitted) … I’ve completely given up now and accepted that this is my life, just as long as it doesn’t get any worse. If this is as bad as it gets, I can just about live with it.”

(Participant 4)

“It’s something I experienced before, but the pain returns when I think about something I failed before. That’s why I try to live my life without thinking about anything.”

(Participant 5)

## 4. Discussion

Pain is a subjective, personal experience, and as such, it requires individual management. In this study, we analyzed the experiences of patients with CRPS to better understand their subjective experiences with the disease.

The first theme was “my own non-stereotypical suffering.” The pain experienced by patients with CRPS showed diverse patterns, intensities, frequencies, and causes but was commonly described as unbearably severe pain. The pain was also somehow different from previously experienced pain. In patients with CRPS, unbearable pain occurred irregularly and even at rest, making them incredibly tense at all times. Previous study has also found that patients with CRPS show persistent pain even after treatment and that they often experience fear regarding continued treatment and using their limbs [23]. A study evaluated emotions and perceptions regarding the affected body parts in CRPS [20] and reported that patients expressed strong negative emotions related to their affected body parts and that pain was so severe that they expressed a desire to cut off these body parts despite a risk of further pain and loss of function. Here, patients with CRPS showed different levels of fear depending on the severity of previously experienced and current pain; in serious cases, patients complained of unbearable pain and fear.

Kim et al. [9] investigated the epidemiology of CRPS in Korea and found that there was no difference in the occurrence of CRPS among different seasons. Patients in our study mentioned that the disease affected their whole life; severe pain was caused even by small stimuli, such as a change in the weather or sounds. They reported that it was often difficult to maintain a normal work or school life, as even everyday movements were affected because of pain and fear of pain. Likewise, Kang et al. [11] reported that most patients diagnosed with CRPS in Korea became unemployed and experienced economic difficulties, including problems with paying medical fees, such that they required help to continue working (e.g., jobs with lower physical demands or shorter working hours).

In our study, the symptoms of CRPS were intractable with extreme changes, a gradual spreading of the affected regions, little therapeutic response, and/or disease recurrence despite treatment. Patients had reported significant suffering until they accepted the disease, after which they gradually became accustomed to the pain. The path to acceptance is not smooth and requires patience, motivation, and strength [19]. These patients require support to muster the strength to continue living and maintain or improve their quality of life. In addition to psychological support, education on a range of coping tools and strategies for use in real-life circumstances should be provided to patients.

The second theme was “complex emotions caused by physical pain.” While patients wanted people to recognize their emotional and mental anguish secondary to physical pain, they also experienced a conflicting desire to hide their diagnosis of CRPS. This is because the participants were misunderstood as strange people or experienced negative gazes due to their diagnosis. Patients with chronic pain require mental and psychological support, as they often exhibit features of generalized anxiety, panic, social anxiety, posttraumatic stress, and/or obsessive-compulsive disorder [24]. In the context of CRPS, we found that individuals close to the patients, including family members and doctors, knew little about the disease, which exacerbated the patients’ subjective difficulties. Most patients in our study were unable to determine the cause of their pain before diagnosis and visited various medical institutions, including traditional Korean medicine clinics, before eventually visiting a pain clinic. In Korea, the mean time from symptom onset to CRPS diagnosis has been reported to be from one month to one year [25]. Additionally, patients in our study showed increasing dissatisfaction with medical personnel over time. Patients with CRPS are increasingly engaged in lawsuits for negligence against medical personnel [25] such that disease education is essential for these staff members. Additionally, because CRPS pain generally exceeds the extent of the responsible injury, it is important to acknowledge patients’ subjective experiences of pain rather than viewing it as an exaggeration and to provide active intervention. Moreover, negative preconceptions about CRPS should be addressed by providing accurate information via the internet or TV programs, which are the main sources of information for these patients. As the patients may feel confused and helpless on the diagnosis of CRPS, it is essential to provide education about the syndrome and its management [26]. Online support and other forms of social support can further help patients feel less socially isolated [18], ultimately improving symptom management and the quality of life of these patients.

Family is an important source of support for patients with CRPS. Yet, 57% of families of CRPS patients reported feeling burdened, and 53% reported experiencing negative emotions as a result of the CRPS diagnosis [27]. Therefore, it is crucial to maintain the psychological stability and health of patients’ families as well. Caregiver involvement is also important; there is a need for caregiver guidelines to specify optimal methods for providing support to CRPS patients, such as active listening and communication techniques [24]. Most patients were amidst litigation, and patients and family members experienced an economic burden because they did not receive adequate support for medical fees. Thus, economic support and other forms of practical support are also needed.

The third theme identified in this study was “living carefully, enduring alone”. Each patient had adopted different coping methods to address CRPS symptoms, but all of them sought methods to help them manage CRPS by themselves and led careful lives to avoid causing pain. Seeking independent coping methods can give the impression of a positive or successful adaptation to the disease. Rodham et al. [19] explored patients’ experience of the transition from an intensive two-week in-patient rehabilitation program to their home environment, where rehabilitation programs play a role in encouraging self-management but not conducive to patient coping, which could result in a negative outcome. Therefore, advice on recovery-supportive environments after discharge is necessary. Moreover, it is vital to assess whether the coping methods used by patients are alleviating pain and helping rehabilitation.

Studying individuals who experience complex phenomena such as pain can provide a multidimensional understanding of pain within the context of physiology, psychology, and social aspects while also helping to develop all areas of medicine and related research [28]. The complex and subjective nature of the pain experience and the effects of personal characteristics on a patient’s capacity to cope with chronic pain indicate that the understanding and management of pain can also be subjective [29]. This makes pain a subjective experience that is not always understood by others. In Korea, several self-help groups are available for rare, intractable diseases, and some of the patients in our study reported that they gained strength and comfort from interactions with these groups or other patients with CRPS. In the United Kingdom, special guidelines have been developed for the diagnosis and management of CRPS; specialist pain management and rehabilitation services are available throughout the country, and symptoms are managed through facilities that include self-help or support groups to maximize physical, psychological, and social functions [30]. Pain control and functional recovery in patients with CRPS are possible in a comprehensive and multidisciplinary environment; however, comprehensive management is still lacking in Korea [9]. Therefore, we must promote cooperation among various institutions for the better management of CRPS.

### Limitations

Our study had several limitations. First, this study was conducted in Korea, and all included patients were Koreans; as such, the experiences described herein might differ from those of patients in other countries and of other ethnicities or cultural backgrounds. Second, our results only address the experiences of adults currently receiving hospital treatment and did not include those of children or adolescents with CRPS or of patients who did not seek medical treatment at a hospital. Despite these limitations, we consider our study to be valuable: there is a lack of research on CRPS in Korea, and although it is difficult to fully understand patient experiences, this study facilitates a deeper understanding of CRPS.

## 5. Conclusions

We performed a thematic analysis to investigate the experiences of adult patients with CRPS in Korea. Three main themes were derived from this study: “my own non-stereotypical suffering”, “complex emotions caused by physical pain”, and “living carefully, enduring alone.” Patients endured internal difficulties alone while trying to find their own methods to alleviate pain, but they required mental, psychological, and social support. Therefore, it is important to educate medical personnel, family members, and caregivers so that patients can receive the appropriate support, maintain employment, obtain sufficient pain control and rehabilitation, and preserve their quality of life.

CRPS remains a poorly understood disease even for medical personnel. Our results can be utilized for the better education of medical personnel to provide a deeper understanding of patients’ experiences with CRPS. Our study outlines important concepts for caregiver programs and can be utilized for the development of awareness and educational programs to change the public perception of CRPS. Our findings also call for the development of various programs, including coping strategies, rehabilitation programs, and social adjustment programs, for patients with CRPS.

## Figures and Tables

**Table 1:** Demographic data of the participants (*n* = 11).

No	Sex	Age (Years)	Marital Status	Offspring	Date of Symptom Onset	Cause	Numeric Rating Scale of Usual Pain
1	F	60	Married	3 daughters, 1 son	October 2005	Fracture	5
2	F	51	Married	1 son	December 2006	Road traffic accident	7
3	F	51	Married	1 daughter	November 2005	Road traffic accident	9
4	M	33	Married	None	November 2006	Road traffic accident	4
5	M	22	Unmarried	None	February 2009	Military training	7
6	M	23	Unmarried	None	March 2009	Intravenous injection	3
7	F	53	Married	2 sons	May 2007	Road traffic accident	8
8	M	39	Married	1 daughter	February 2008	Road traffic accident	7
9	M	54	Married	None	2007	Surgery in 1988	8
10	F	39	Unmarried	None	August 2003	Neurosurgery	3
11	M	49	Married	1 daughter, 1 son	March 2009	Road traffic accident	6

## Data Availability

The data that support the findings of this study are available from the corresponding author upon reasonable request.
